# Seasonal Food Insecurity in Haydom, Tanzania, Is Associated with Low Birthweight and Acute Malnutrition: Results from the MAL-ED Study

**DOI:** 10.4269/ajtmh.18-0547

**Published:** 2019-01-02

**Authors:** Elizabeth T. Rogawski McQuade, Stephen Clark, Eliwaza Bayo, Rebecca J. Scharf, Mark D. DeBoer, Crystal L. Patil, Jean C. Gratz, Eric R. Houpt, Erling Svensen, Estomih R. Mduma, James A. Platts-Mills

**Affiliations:** 1 Department of Public Health Sciences, University of Virginia, Charlottesville, Virginia;; 2 Division of Infectious Diseases and International Health, University of Virginia, Charlottesville, Virginia;; 3 Global Health Research Centre, Haydom Lutheran Hospital, Manyara Region, Tanzania;; 4 Department of Pediatrics, University of Virginia, Charlottesville, Virginia;; 5 Department of Women, Children and Family Health Science, College of Nursing, University of Illinois at Chicago, Illinois;; 6 Haukeland University Hospital, Bergen, Norway

## Abstract

In rural agricultural communities in Africa, particularly those with a single annual harvest, the preharvest period has been associated with increased food insecurity. We estimated the association between seasonal food insecurity and childhood malnutrition in Haydom, Tanzania. Children enrolled in a birth cohort study were followed twice weekly to document food intake and monthly for anthropometry until the age of 2 years. Household food insecurity was reported by caregivers every 6 months. We modeled the seasonality of food insecurity and food consumption, and estimated the impact of birth season on enrollment weight and subsequent malnutrition. Finally, we described the seasonality of admissions for acute malnutrition at a local referral hospital (Haydom Lutheran Hospital) from 2010 to 2015. Food insecurity was highly seasonal, with a peak from December to February. Children born during these 3 months had an average 0.35 *z*-score (95% CI: 0.12, 0.58) lower enrollment weight than children born in other months. In addition, weight-for-length *z*-scores measured in these months were on average 0.15 *z*-scores lower (95% CI: 0.10, 0.20) than that in other months, adjusting for enrollment weight and seasonal infectious diseases, and this disparity was sustained up to the age of 2 years. Correspondingly, the number of admissions with acute malnutrition at the local hospital was highest at this time, with twice as many cases in December–February compared with June–August. We identified acute and chronic malnutrition associated with seasonal food insecurity and intake. Targeting of prenatal care and child-feeding interventions during high food insecurity months may help reduce child malnutrition.

## INTRODUCTION

Food insecurity refers to uncertain access to sufficient food for healthy growth and development,^1^ and can manifest in myriad ways: reduced intake of food or preferred foods, sale of assets or seeking additional work to pay for food, movement to another area, and even a reduction in the number of household dependents.^2^ In agricultural communities, food insecurity is commonly seasonal, resulting in a preharvest “hunger season” with high risk for reduced nutritional status, psychological distress, and increased infectious disease at times of increased workloads.^3,4^ In areas with only one harvest per year, the preharvest season is a vulnerable period with potentially lasting impacts on health outcomes.^5–7^ Young children are particularly susceptible to the deleterious effects of seasonal food insecurity, including increased mortality,^8^ because the first 2 years of life are a critical period for child growth and development with lasting impact into adulthood.^9^

Haydom is a small town in the Manyara region of Tanzania. The area is ethnically and geographically diverse and relies primarily on subsistence agriculture.^10^ Crop yields and the resulting availability of food are dependent on the duration of the short (November and December) and long rains (January to May), and there is only one annual harvest following the rainy season. Food insecurity has been previously characterized in Haydom^11^ and elsewhere in rural Tanzania,^3^ and has been linked to food availability that is dependent on the agricultural season. There is very low malaria transmission in the region because of high altitude.^12,13^

The seasonality of child growth has also been explored in other regions of Africa, including in Malawi^14^ and the Gambia,^6^ where deficits in weight and height were larger in the rainy season. However, the association of seasonal food insecurity with child health outcomes has not been quantified in Tanzania. We assessed seasonal patterns of food insecurity, intake of foods, birthweight, and measures of malnutrition among children in Haydom, Tanzania, to estimate the impact of the high food insecurity season on these outcomes. We also assessed the seasonality of severe outcomes from pediatric admissions with acute malnutrition at the local hospital.

## MATERIALS AND METHODS

The Etiology, Risk Factors, and Interactions of Enteric Infections and Malnutrition and the Consequences for Child Health and Development (MAL-ED) study design and methods,^15^ and the site in Haydom, Tanzania,^10^ have been previously described. Ethical approval was obtained from the National Institute for Medical Research in Tanzania and the Institutional Review Board of the University of Virginia. Written informed consent was obtained from the parent or guardian of every child. Briefly, children were enrolled within 17 days of birth and followed until the age of 2 years. Child food intake was recorded by a 24-hour food recall and illnesses were recorded by a maternal report at twice-weekly home visits. Diarrhea was defined as maternal report of three or more loose stools in 24 hours or one stool with visible blood. Acute lower respiratory infection (ALRI) was defined as cough or shortness of breath with a rapid respiratory rate determined by fieldworkers (defined by the average of two measurements per day that were > 60 breaths per minute when the child was < 2 months old; > 50 breaths per minute at age 2 months to 1 year; and > 40 breaths per minute at age ≥ 1 year).^16^ Anthropometry was measured monthly and converted into weight-for-age *z*-scores (WAZ), length-for-age *z*-scores (LAZ), and weight-for-length *z*-scores (WLZ) using the 2006 World Health Organization (WHO) child growth standards.^17^

Household food insecurity was assessed every 6 months with the question, “In the past 4 weeks, did you worry that your household would not have enough food?” We considered any frequency of worry (rarely, sometimes, or often) in response to this question as a report of food insecurity because responses of “sometimes” or “often” were uncommon (7.5% and 1.1%, respectively). Despite the subjectivity of this measure, the question was asked in the same way and in the same population over time such that relative differences by season are meaningful. Socioeconomic status (SES) was summarized as a score based on water access, assets, maternal education, and income and was averaged over four biannual surveys.^18^

Haydom Lutheran Hospital (HLH) is a rural 450-bed referral hospital situated in the town closest to the MAL-ED study area. The hospital has a catchment area of 74 villages and towns and serves approximately two million people,^19,20^ including all children in the Haydom MAL-ED cohort. We reviewed all hospital discharges from January 2010 to December 2015 among children under the age of five years for diagnoses of malnutrition (defined as malnutrition, acute malnutrition, severe acute malnutrition, kwashiorkor, or marasmus), diarrhea (defined as gastroenteritis, diarrhea, dysentery, acute watery diarrhea, giardiasis, or amebiasis), ALRIs (pneumonia), and all other diagnoses. Age, gender, and mortality associated with these admissions were also collected.

### Data analysis.

The seasonality of the prevalence of food insecurity was modeled using Poisson regression for the number of reports per month. Highly variable crude monthly rates across the years of the study period were smoothed with linear and quadratic terms for the month of the year (*m*), and the terms sin (2π*m*/12), cos (2π*m*/12), sin (4π*m*/12), and cos (4π*m*/12) based on optimal fit by the Akaike information criterion. We modeled child food intake patterns using log binomial regression for the intake (yes/no) of certain foods by month. We modeled diarrhea and ALRI incidence by calendar month using pooled logistic regression for incident episodes from birth to the age of 2 years. We similarly assessed the seasonality of anthropometric outcomes by using linear regression to model average WLZ, WAZ, and LAZ by month.

To estimate differences in food insecurity, food intake, and anthropometry across seasons, months were split into quarters that capture variation in food insecurity: December–February, March–May, June–August, and September–November. We used general estimating equations and robust variance to account for correlation between measurements within children, and adjusted for the incidence of seasonal infectious diseases: diarrhea and ALRI. Heterogeneity by gender, SES score, and number of siblings was assessed by the likelihood ratio test.

We assessed long-term disparities in child health based on seasonal birth cohorts. We used linear regression to estimate the associations between season of birth and WAZ at enrollment and WAZ, LAZ, and WLZ at age 2 years.

The seasonality of malnutrition-related and other admissions to HLH and mortality among children aged less than 5 years were modeled using Poisson regression for the total number of cases per month and the number of cases stratified by gender to assess the relative rate of admissions and mortality by season. The seasonality of diagnosis-specific case fatality rates was modeled using Poisson regression for the number of deaths per month with an offset for the number of diagnosis-specific admissions in that month. Analyses with the subset of admissions among children less than the age of two years were consistent with the analysis of all children less than the age of five years (not shown).

## RESULTS

### Observational community-based cohort.

Among 262 children enrolled at the Haydom, Tanzania, MAL-ED site, 211 children were followed up until the age of 2 years between 2009 and 2013. The median enrollment age was 7 days (IQR: 5, 9) and the mean WAZ at enrollment was −0.16 (SD = 0.91). At the age of 2 years, the mean WAZ was −1.33 (SD = 1.01), with 23.0% (*n* = 29) of children underweight (WAZ < −2). Prevalence of stunting was 16.4% (*n* = 43) at enrollment (mean LAZ = −1.04; SD: 1.17) and increased to 70.6% (*n* = 149) age 2 years (mean LAZ = −2.66; SD: 1.01), with nearly half of these being severely stunted (LAZ < −3; *n* = 72; 34.1% of all children). The mean WLZ at 2 years was 0.07 (SD: 0.98), and no children were wasted (WLZ < −2).

**Figure 1.**
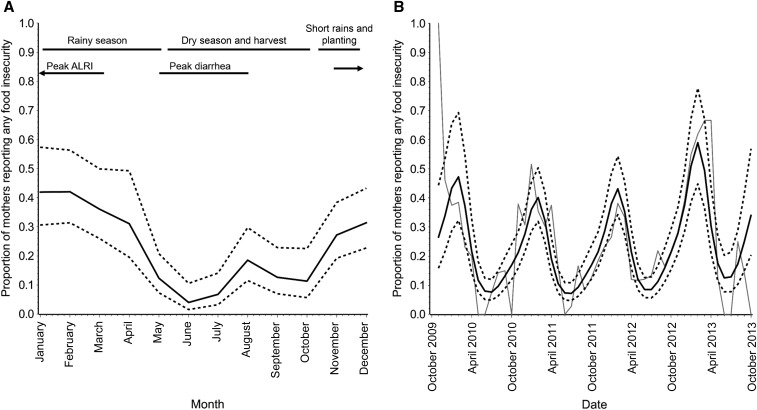
Prevalence of food insecurity by calendar month (**A**) and across years of the study from 2009 to 2013 (**B**) among 262 children at the Haydom, Tanzania, site of the MAL-ED study. (**A**) Prevalence (solid line) and 95% confidence limits (dotted lines); (**B**) modeled prevalence (solid black line) with 95% confidence limits (dotted lines) overlaid on crude prevalence (solid gray line). ALRI = acute lower respiratory infection.

A median number of five food insecurity questionnaires were conducted for each household at 6-month intervals over the child’s first 2 years. Caregivers reported at least some food insecurity during 23.0% of these questionnaires (*n* = 268 of 1,163 total), and food insecurity was reported occasionally for most households in the community (60.3% reported food insecurity at least once; Table 1). However, the prevalence was highly seasonal, with the highest proportion of mothers reporting food insecurity in December through February (Figure 1A). Prevalence during these months was 2.37 (95% CI: 1.94, 2.88) times the prevalence during the rest of the year. This pattern was consistent across the years of the study from 2009 to 2013, although the magnitude of peak insecurity varied (Figure 1B).

**Table 1:** Prevalence of food insecurity by demographic factors among 262 children in the Haydom, Tanzania, MAL-ED site

	No. of food insecurity questionnaires	No. (%) of questionnaires reporting food insecurity	Food insecurity prevalence ratio (95% CI)*
Season			
June, July, and August	297	28 (9.4)	1.0
September, October, and November	276	51 (18.5)	1.92 (1.25, 2.97)
March, April, and May	272	68 (25.0)	2.61 (1.76, 3.88)
December, January, and February	318	121 (38.1)	3.99 (2.88, 5.54)
SES			
4th quartile	319	98 (30.7)	1.0
3rd quartile	286	73 (25.5)	1.63 (1.04, 2.56)
2nd quartile	275	60 (21.8)	1.92 (1.26, 2.92)
1st quartile	283	37 (13.1)	2.23 (1.48, 3.36)
Number of children†			
1–2	306	57 (18.6)	1.0
3–5	522	122 (23.4)	1.28 (0.96, 1.70)
6+	332	89 (26.8)	1.41 (1.04, 1.90)

SES = socioeconomic status.

* Estimates adjusted for season and SES.

† Missing for three questionnaires.

There was heterogeneity in food insecurity prevalence by SES and other demographics. Adjusting for seasonality, households in the bottom quartile of the SES score were more than twice as likely to report food insecurity compared with households in the top quartile of the SES score (Table 1). Furthermore, the number of children the mother had was also associated with food insecurity. Adjusting for seasonality and SES, mothers with three to five children had a 27.8% higher prevalence (95% CI: −3.7, 69.6) of food insecurity and mothers with six or more children had a 40.8% (95% CI: 4.3, 89.9) higher prevalence of food insecurity compared with mothers with only one or two children (Table 1).

Reported child food intake patterns trended with the seasonality of food insecurity for several foods (Supplemental Figures 1 and 2). Although maize was almost universally available and consumed by children throughout the year, animal milk consumption matched the seasonality of food insecurity, with 8.7% fewer caregivers (95% CI: 5.3, 12.0) reporting their child consumed animal milk in December–February compared with the rest of the year. Consumption of some foods was almost completely restricted to certain seasons. For example, root vegetables were mainly available from March to November, with consumption 76.8% lower (95% CI: 71.5, 81.1) in December through February. Similarly, fermented milk was only available from July to October, with < 1% of 24-hour food recalls, including fermented milk, in December–February.

Caregiver report of food insecurity was responsive to acute changes in food availability as reflected by child consumption patterns. For example, in January–April 2013, there was an acute correlation between food insecurity and food consumption demonstrated by a peak prevalence of food insecurity of 66.7% and a coincident sharp drop in the proportion of children consuming animal milk during this time (from 74.6% consuming animal milk on average in October–December 2012 to 56.5% in January 2013; Supplemental Figure 3).

Children in the cohort experienced 628 diarrhea episodes and 133 ALRI episodes over their first 2 years of life. The incidences of diarrhea (Supplemental Figure 4) and ALRI (Supplemental Figure 5) were variable over the months of the year such that seasonality was less clear. The peak diarrhea season was in May–July (209 episodes; 33.3%) and ALRI cases were most frequent in November–December (36 episodes; 27.1%), with smaller peaks in February, June, and August.

**Figure 2.**
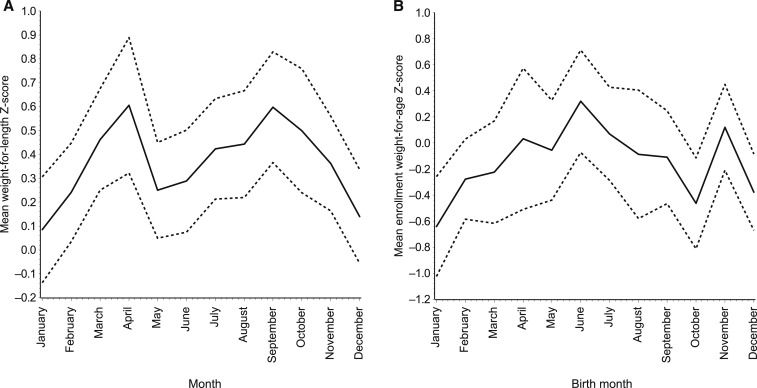
(**A**) Mean (solid line) and 95% confidence limits (dotted lines) of weight-for-length *z*-score by calendar month (**A**) and mean weight-for-age Z-score at enrollment (within 17 days of birth) by calendar month (**B**) among 262 children at the Haydom, Tanzania, site of the MAL-ED study.

The measures of seasonal variation in food insecurity and food intake were further reflected in the anthropometric measurements of the children, and were not explained by the incidence of seasonal infectious diseases. Weight-for-length *z*-scores, which can indicate acute undernutrition or wasting (defined as WLZ < −2), were measured monthly from 1 to 24 months of age and were 0.15 *z*-scores lower (95% CI: 0.10, 0.20) in December–February than the rest of the year, adjusting for enrollment weight, and any diarrhea and ALRI during the month of anthropometric measurement (Figure 2A). There were no differences in these associations by gender (*P* for heterogeneity = 0.8), SES score (*P* = 0.2), or number of children (*P* = 0.2).

The impact of seasonal food insecurity was most striking among quarterly birth cohorts. Children born in December through February (*n* = 85) had an average of 0.35 (95% CI: 0.12, 0.58) lower enrollment WAZ than children born in other months (Figure 2B). Among the subset of 132 children with measured birthweight, this association was nearly equivalent (0.37 *z*-score decrement, 95% CI: 0.00, 0.73). This disparity was diminished but still apparent at the age of 2 years for WAZ, LAZ, and WLZ metrics, when adjusting for the incidence of seasonal illnesses, diarrhea, and ALRI (Table 2).

**Table 2:** Associations between birth season and anthropometric outcomes among 262 children in the Haydom, Tanzania, MAL-ED site

Food insecurity prevalence	Birth season	Enrollment WAZ	WAZ at 24 months	LAZ at 24 months	WLZ at 24 months
		Difference (95% CI)*			
Lowest	June, July, and August	0	0	0	0
Intermediate	September, October, and November	−0.26 (−0.58, 0.05)	−0.09 (−0.47, 0.29)	−0.22 (−0.60, 0.16)	0.03 (−0.34, 0.40)
Intermediate	March, April, and May	−0.23 (−0.57, 0.12)	−0.16 (−0.57, 0.24)	−0.42 (−0.84, −0.01)	0.03 (−0.37, 0.43)
Highest	December, January, and February	−0.53 (−0.83, −0.22)	−0.29 (−0.66, 0.08)	−0.30 (−0.67, 0.07)	−0.17 (−0.53, 0.19)

LAZ = length-for-age *z*-scores; WAZ = weight-for-age *z*-score; WLZ = weight-for-length *z*-score.

* Estimates for anthropometry at 24 months are adjusted for number of diarrhea and acute lower respiratory infection episodes in the first 2 years of life.

### Haydom Lutheran Hospital admissions.

These high-resolution birth cohort data also matched trends from HLH over a similar time period from 2010 to 2015. During this period, there were 11,266 total admissions to the hospital among children less than the age of five years, with 559 (5.0%) of them for malnutrition-related diagnoses, 2,982 (26.5%) for diarrhea, 3,001 (26.5%) for ALRI, and 4,839 (43.0%) for other diagnoses. The majority of these children were less than the age of two years (*n* = 9,374/11,266, 83.2%).

Of the children admitted for malnutrition, the majority (*n* = 475, 85.0%) were diagnosed with malnutrition alone, whereas 19 were co-diagnosed with diarrhea (3.4%), 22 (3.9%) were co-diagnosed with ALRI, and 43 (7.7%) were co-diagnosed with something else. 56.2% of children less than five years of age admitted with malnutrition were male (*n* = 314).

**Figure 3.**
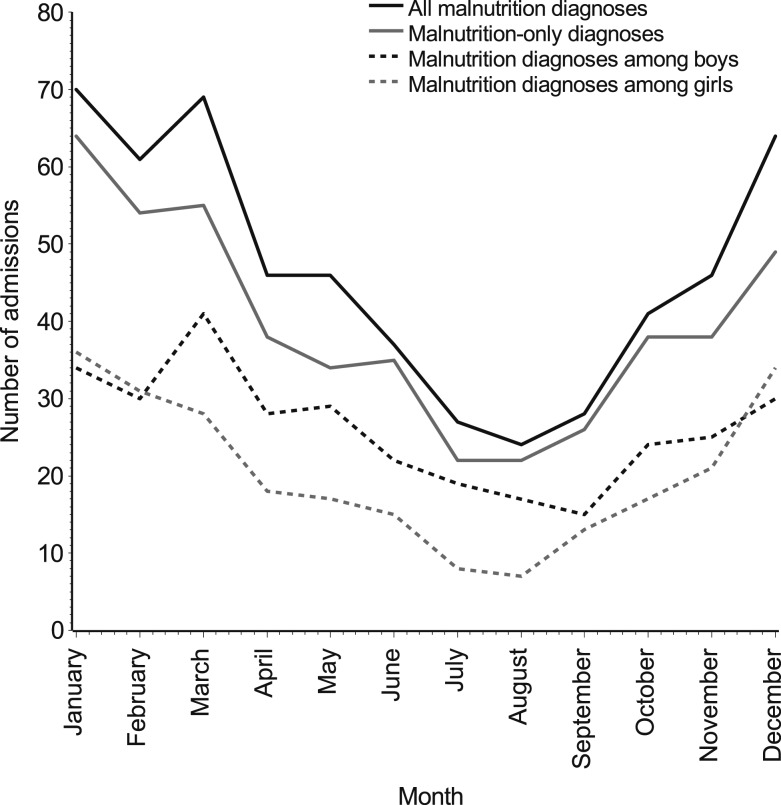
Number of malnutrition-related admissions among children less than the age of five years by calendar month at Haydom Lutheran Hospital from 2010 to 2015. Admission numbers are presented for all malnutrition diagnoses (black solid line), malnutrition-only diagnoses (without any co-diagnoses; gray solid line), malnutrition diagnoses among boys (black dotted line), and malnutrition diagnoses among girls (gray dotted line).

The number of malnutrition-related admissions was 60.7% higher (incidence rate ratio [IRR]: 1.61, 95% CI: 1.37, 1.93) from December to February than that in all other months, and twice as high in these months than in June–August (IRR: 2.22, 95% CI: 1.72, 2.85). This seasonality was present even when excluding admissions with co-diagnoses (Figure 3). Although malnutrition-related admissions were more common among boys, the seasonality of admissions was more pronounced among girls. Girls were more than twice as likely to be admitted from December to February than in June–August (IRR: 2.10, 95% CI: 1.63, 2.71), whereas the IRR was only 1.28 (95% CI: 1.01, 1.63) among boys.

**Figure 4.**
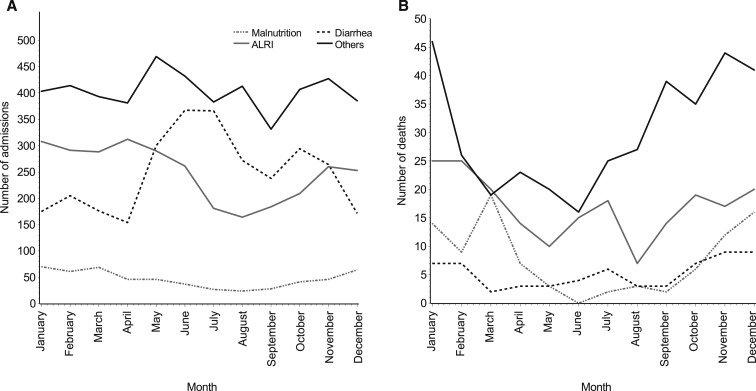
Number of admissions (**A**) and deaths (**B**) by diagnosis and calendar month among children less than the age of five years at Haydom Lutheran Hospital from 2010 to 2015. ALRI = acute lower respiratory infection.

The seasonality of admissions for ALRI was similar to that for malnutrition, with peak cases in January to April (Figure 4A). Conversely, the seasonality of admissions for diarrhea was the opposite of that for malnutrition, such that peak admissions for diarrhea were in June to October. There was no clear seasonality for other diagnoses.

The overall mortality rate was 6.3% (*n* = 710) for all admissions, and the mortality rate for malnutrition-related diagnoses was much higher than that for other diagnoses. The mortality rate for malnutrition with no other diagnoses was 14.9% (71/475), for malnutrition with a co-diagnosis was 26.2% (22/84), for diarrhea was 2.1% (63/2,982), for ALRI was 6.8% (204/3,001), and for other diagnoses was 7.5% (361/4,839).

The seasonality of mortality in terms of absolute numbers of deaths was striking and similar for malnutrition, ALRI, and other diagnoses (Figure 4B). Accounting for monthly number of admissions, the case fatality rate for malnutrition was also seasonal with highest rates in November through March (Supplemental Figure 6). Among 93 deaths in 559 children less than the age of five years with a malnutrition-related diagnosis, only five occurred from June to August, resulting in a malnutrition case fatality rate during the rest of the year that was more than three times that in June–August (IRR: 3.29, 95% CI: 1.34, 8.10). Interestingly, the case fatality rates for diarrhea matched the seasonality of malnutrition, despite the opposite seasonality pattern for diarrhea incidence (resulting in peak incidence in months with the lowest case fatality). Children with diarrhea in the high food insecurity months (December–February) had more than double the case fatality rate than children with diarrhea during the rest of the year (IRR: 2.54, 95% CI: 1.52, 4.24). A similar pattern was observed for case fatality rates for ALRI and other diagnoses, although on a smaller magnitude. The case fatality rate was 32% higher (IRR: 1.32, 95% CI: 0.99, 1.76) and 38% higher (IRR: 1.38, 95% CI: 1.10, 1.72) in December–February for ALRI and other diagnoses, respectively.

## DISCUSSION

Food insecurity in Haydom, Tanzania, was seasonal and was reflected in child food intake and concurrent measures of acute malnutrition. The seasonality was more pronounced in households of low SES and with a higher number of dependents. The association between increased food insecurity and lower enrollment and birthweights suggests an in utero effect of reduced food availability in the hunger season, most importantly in the third trimester of pregnancy. These results correspond to the findings from the Dutch Famine Birth Cohort Study, which showed a decrement in birthweight of 250 g among infants whose mothers were undernourished in the third trimester due to the Dutch Hunger Winter of 1944–1945.^21^ Because our main analysis was conducted using enrollment weight, greater susceptibility to weight loss in the first 2 weeks of life among children born in the hunger season may also contribute to the seasonal differences and may be explained by low quantity and/or quality of breast milk during this time. These disparities were persistent and associated with smaller but sustained deficits at the age of 2 years, which suggests incomplete catch-up growth among children born in the high food insecurity season.

Differences in severe outcomes were even more disparate by season as demonstrated by admissions for malnutrition and related mortality at HLH. Interestingly, the hospital admissions showed gender differences, such that admissions of girls had larger seasonal variation, despite lower admission rates than for boys overall. A higher burden of malnutrition among boys than in girls has been previously noted, especially in sub-Saharan Africa.^22–25^ However, the heightened seasonality among girls suggested that they may be especially vulnerable to fluctuations in food availability. The analyses of diarrhea and ALRI in the birth cohort and hospital data suggest that other seasonal illnesses do not explain the seasonality of anthropometric measures. Interestingly, case fatality rates for these and other diagnoses at the hospital varied with the seasonality of malnutrition, which suggests that these illnesses are more severe in the hunger season, and underlying (but not necessarily diagnosed) malnutrition may be contributing to poor outcomes.

The high food insecurity months correspond with the agricultural cycle in Haydom. November or December marks the beginning of the short rains, field preparations, and the dwindling of maize stores. Long rains begin in January, and planting occurs in the rainy season through March. Food stores dwindle during this time, whereas the cost of food increases.^26^ The harvest of beans and then maize occurs in March and May to July, respectively, which leads to sufficient availability of foods. The seasonality of food availability is well understood by families in this region, but the impact on child health outcomes has not been fully appreciated.

The seasonality of malnutrition has implications for research of other seasonal phenomena among children in low-resource settings, especially seasonal infectious diseases. There may be confounding results by season and/or birth month, which should be explored and taken into account if present. The randomness of birth month may also be used as an instrumental variable to assess relationships between risk factors and outcomes of malnutrition.

This study was limited by relatively infrequent assessment of food insecurity every 6 months. Although participants were enrolled throughout the year such that we have robust cohort-level data on food insecurity during each month, we were unable to associate food insecurity and anthropometry at the individual level. In addition, we did not have information about seasonal maternal illnesses during pregnancy that could affect birth size. However, the very low prevalence of malaria in this region due to high altitude^12,13^ suggests that malaria in pregnancy is not a main driver of seasonal changes in birthweight. We also did not have measurement of preterm birth, and birthweight was only measured for half of the enrolled children. However, birthweights were highly correlated with enrollment weights (Pearson correlation coefficient: 0.69), and the seasonality results were nearly equivalent when using either enrollment weight or birthweight. Finally, although there were not enough hospitalizations within the MAL-ED cohort (*n* = 86) for seasonal analysis, the analysis of all hospitalizations at the hospital to which the cohort children were referred provides relevant information on severe outcomes in this population.

These results suggest that children born in the “hunger season” are especially vulnerable to poor health outcomes. The implications may extend into adulthood as severe malnutrition and poor growth have been associated with reduced cognitive function^27,28^ and chronic diseases later in life.^29–31^ Prenatal care interventions, including nutritional supplementation, could be targeted during high food insecurity months to reduce seasonal disparities. Energy and protein dietary supplementation and nutritional education for pregnant women have shown to be effective^32^ and are recommended by WHO with the acknowledgment that local context, including the seasonality of food availability, should be taken into account when designing interventions.^33^ Similarly, targeting of child-feeding interventions in the community and/or hospital setting during the high food insecurity season may have the greatest impact on reducing child malnutrition and mortality. In low-resource communities that rely heavily on local agriculture, vulnerability to seasonal availability of food will only increase with climate change.^1^ Increased and potentially targeted vigilance to prevent young children at highest risk from having poor growth and development outcomes related to undernutrition is needed.

## Supplementary Files

Supplemental figures

## References

[1] FAO, IFAD, UNICEF, WFP, WHO, 2017 The State of Food Security and Nutrition in the World 2017. Building Resilience for Peace and Food Security. Rome, Italy: FAO.

[2] Shipton P, 1990 African famines and food security: anthropological perspectives. Annu Rev Anthropol 19: 353–394.

[3] Hadley C, 2005 Ethnic expansions and between-group differences in children’s health: a case study from the Rukwa Valley, Tanzania. Am J Phys Anthropol 128: 682–692. 15895429 10.1002/ajpa.20056

[4] Hadley C Patil CL, 2006 Food insecurity in rural Tanzania is associated with maternal anxiety and depression. Am J Hum Biol 18: 359–368. 16634017 10.1002/ajhb.20505

[5] Roba KT O’Connor TP Belachew T O’Brien NM, 2016 Variations between post- and pre-harvest seasons in stunting, wasting, and Infant and Young Child Feeding (IYCF) practices among children 6–23 months of age in lowland and midland agro-ecological zones of rural Ethiopia. Pan Afr Med J 24: 163. 27795761 10.11604/pamj.2016.24.163.9387 PMC5072826

[6] Nabwera HM Fulford AJ Moore SE Prentice AM, 2017 Growth faltering in rural Gambian children after four decades of interventions: a retrospective cohort study. Lancet Glob Health 5: e208–e216. 28104187 10.1016/S2214-109X(16)30355-2 PMC5340725

[7] Branca F Pastore G Demissie T Ferro-Luzzi A, 1993 The nutritional impact of seasonality in children and adults of rural Ethiopia. Eur J Clin Nutr 47: 840–850. 8156981

[8] Moore SE Cole TJ Poskitt EM Sonko BJ Whitehead RG McGregor IA Prentice AM, 1997 Season of birth predicts mortality in rural Gambia. Nature 388: 434. 9242401 10.1038/41245

[9] Guerrant RL DeBoer MD Moore SR Scharf RJ Lima AAM, 2013 The impoverished gut—a triple burden of diarrhoea, stunting and chronic disease. Nat Rev Gastroenterol Hepatol 10: 220–229. 23229327 10.1038/nrgastro.2012.239 PMC3617052

[10] Mduma ER 2014 The etiology, risk factors, and interactions of enteric infections and malnutrition and the consequences for child health and development study (MAL-ED): description of the Tanzanian site. Clin Infect Dis 59 (Suppl 4): S325–S330. 25305305 10.1093/cid/ciu439

[11] Hanselman B Ambikapathi R Mduma E Svensen E Caulfield LE Patil CL, 2018 Associations of land, cattle and food security with infant feeding practices among a rural population living in Manyara, Tanzania. BMC Public Health 18: 159. 29351750 10.1186/s12889-018-5074-9 PMC5775554

[12] Drakeley CJ Carneiro I Reyburn H Malima R Lusingu JPA Cox J Theander TG Nkya WMMM Lemnge MM Riley EM, 2005 Altitude-dependent and-independent variations in *Plasmodium falciparum* prevalence in northeastern Tanzania. J Infect Dis 191: 1589–1598. 15838785 10.1086/429669

[13] DeBoer MD 2018 Early Life Interventions for Childhood Growth and Development in Tanzania (ELICIT): a protocol for a randomised factorial, double-blind, placebo-controlled trial of azithromycin, nitazoxanide and nicotinamide. BMJ Open 8: e021817. 10.1136/bmjopen-2018-021817 PMC6042604 29982218

[14] Maleta K Virtanen SM Espo M Kulmala T Ashorn P, 2003 Seasonality of growth and the relationship between weight and height gain in children under three years of age in rural Malawi. Acta Paediatr 92: 491–497. 12801119 10.1111/j.1651-2227.2003.tb00584.x

[15] MAL-ED Network Investigators, 2014 The MAL-ED study: a multinational and multidisciplinary approach to understand the relationship between enteric pathogens, malnutrition, gut physiology, physical growth, cognitive development, and immune responses in infants and children up to 2 years of age in resource-poor environments. Clin Infect Dis 59 (Suppl 4): S193–S206. 25305287 10.1093/cid/ciu653

[16] Richard SA Barrett LJ Guerrant RL Checkley W Miller MA; MAL-ED Network Investigators, 2014 Disease surveillance methods used in the 8-site MAL-ED cohort study. Clin Infect Dis 59 (Suppl 4): S220–S224. 25305290 10.1093/cid/ciu435 PMC4204606

[17] WHO, 2006 WHO Child Growth Standards: Length/height-for-Age, Weight-for-Age, Weight-for-Length, Weight-for-Height and Body Mass Index-for-Age, Methods and Development. Geneva, Switzerland: World Health Organization Available at: http://www.who.int/childgrowth/standards/Technical_report.pdf?ua=1. Accessed May 9, 2016.

[18] Psaki SR 2014 Measuring socioeconomic status in multicountry studies: results from the eight-country MAL-ED study. Popul Health Metr 12: 8. 24656134 10.1186/1478-7954-12-8 PMC4234146

[19] Evjen-Olsen B Olsen OE Kvåle G, 2009 Achieving progress in maternal and neonatal health through integrated and comprehensive healthcare services—experiences from a programme in northern Tanzania. Int J Equity Health 8: 27. 19642990 10.1186/1475-9276-8-27 PMC2725038

[20] Mduma E Ersdal H Svensen E Kidanto H Auestad B Perlman J, 2015 Frequent brief on-site simulation training and reduction in 24-h neonatal mortality—an educational intervention study. Resuscitation 93: 1–7. 25957942 10.1016/j.resuscitation.2015.04.019

[21] Stein AD Ravelli AC Lumey LH, 1995 Famine, third-trimester pregnancy weight gain, and intrauterine growth: the Dutch famine birth cohort study. Hum Biol 67: 135–150. 7721275

[22] Svedberg P, 1990 Undernutrition in sub‐Saharan Africa: is there a gender bias? J Dev Stud 26: 469–486.

[23] Wells JCK, 2000 Natural selection and sex differences in morbidity and mortality in early life. J Theor Biol 202: 65–76. 10623500 10.1006/jtbi.1999.1044

[24] Wamani H Astrøm AN Peterson S Tumwine JK Tylleskär T, 2007 Boys are more stunted than girls in sub-Saharan Africa: a meta-analysis of 16 demographic and health surveys. BMC Pediatr 7: 17. 17425787 10.1186/1471-2431-7-17 PMC1865375

[25] Condo JU Gage A Mock N Rice J Greiner T, 2015 Sex differences in nutritional status of HIV-exposed children in Rwanda: a longitudinal study. Trop Med Int Health 20: 17–23. 25345559 10.1111/tmi.12406

[26] Kaminski J Christiaensen L Gilbert CL, 2016 Seasonality in local food markets and consumption: evidence from Tanzania. Oxf Econ Pap 68: 736–757.

[27] Adair LS COHORTS Group, 2013 Associations of linear growth and relative weight gain during early life with adult health and human capital in countries of low and middle income: findings from five birth cohort studies. Lancet 382: 525–534. 23541370 10.1016/S0140-6736(13)60103-8 PMC3744751

[28] Sudfeld CR McCoy DC Danaei G Fink G Ezzati M Andrews KG Fawzi WW, 2015 Linear growth and child development in low- and middle-income countries: a meta-analysis. Pediatrics 135: e1266–e1275. 25847806 10.1542/peds.2014-3111

[29] Eriksson JG Forsén T Tuomilehto J Osmond C Barker DJP, 2001 Early growth and coronary heart disease in later life: longitudinal study. BMJ 322: 949–953. 11312225 10.1136/bmj.322.7292.949 PMC31033

[30] Lelijveld N 2016 Chronic disease outcomes after severe acute malnutrition in Malawian children (ChroSAM): a cohort study. Lancet Glob Health 4: e654–e662. 27470174 10.1016/S2214-109X(16)30133-4 PMC4985564

[31] Wells JCK Chomtho S Fewtrell MS, 2007 Programming of body composition by early growth and nutrition. Proc Nutr Soc 66: 423–434. 17637095 10.1017/S0029665107005691

[32] Ota E Hori H Mori R Tobe-Gai R Farrar D, 2015 Antenatal dietary education and supplementation to increase energy and protein intake. Cochrane Database Syst Rev 6: CD000032 DOI: 10.1002/14651858.CD000032.pub3. PMC12634316 26031211

[33] WHO, 2016 WHO Recommendations on Antenatal Care for a Positive Pregnancy Experience. Luxembourg: World Health Organization Available at: http://apps.who.int/iris/bitstream/handle/10665/250796/9789241549912-eng.pdf;jsessionid=9FE5874AC17B3F31F6F42830599E23C8?sequence=1. Accessed June 23, 2018. 28079998

