# Supplementary material for: Seasonal Food Insecurity in Haydom, Tanzania, Is Associated with Low Birthweight and Acute Malnutrition: Results from the MAL-ED Study
Source: Am J Trop Med Hyg. 2019 Jan 2;100(3):681–7. doi: 10.4269/ajtmh.18-0547 (PMC6402900; doi:10.4269/ajtmh.18-0547)
Supplement: Supplementary file 1 [file tpmd180547.SD1.pdf]

Supplemental Material

Seasonal food insecurity in Haydom, Tanzania is associated with low birthweight and acute malnutrition: results from the MAL-ED study

Elizabeth T. Rogawski McQuade*[1,2], Stephen Clark[2], Eliwaza Bayo[3], Rebecca J. Scharf[4], Mark D. DeBoer[4], Crystal L. Patil[5], Jean C. Gratz[2], Eric R. Houpt[2], Erling Svensen[3,6], Estomih R. Mduma[3], James A. Platts-Mills[2]

[1]Department of Public Health Sciences, University of Virginia, Charlottesville, VA, USA
[2]Division of Infectious Diseases & International Health, University of Virginia, Charlottesville, VA, USA
[3]Global Health Research Centre, Haydom Lutheran Hospital, Manyara Region, Tanzania
[4]Department of Pediatrics, University of Virginia, Charlottesville, VA, USA
[5]Department of Women, Children & Family Health Science, College of Nursing, University of Illinois at Chicago, IL, USA
[6]Haukeland University Hospital, Bergen, Norway

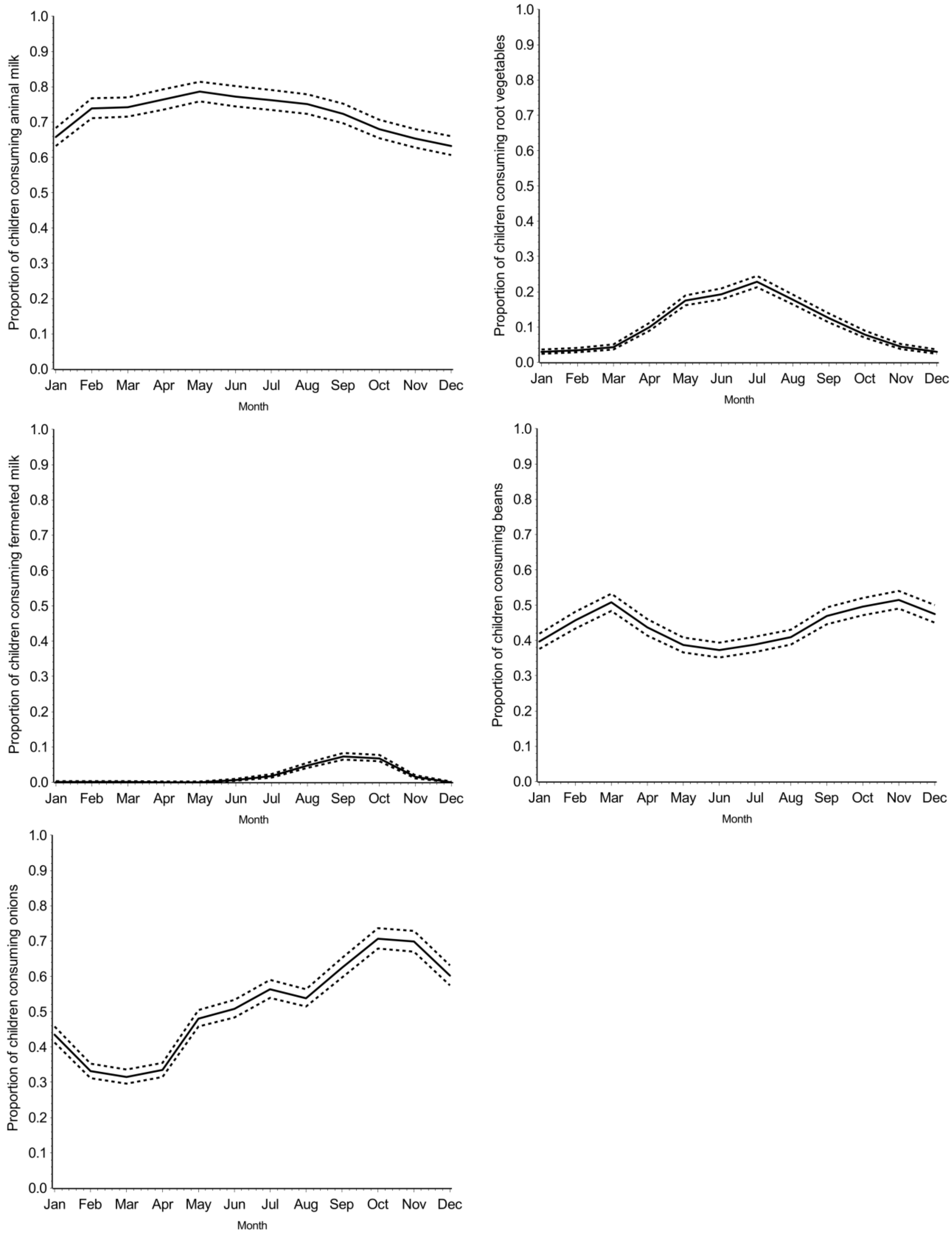

Figure S1. Child intake of seasonal foods by 24-hour food recall by calendar month among 262 children at the Haydom, Tanzania site of the MAL-ED study.

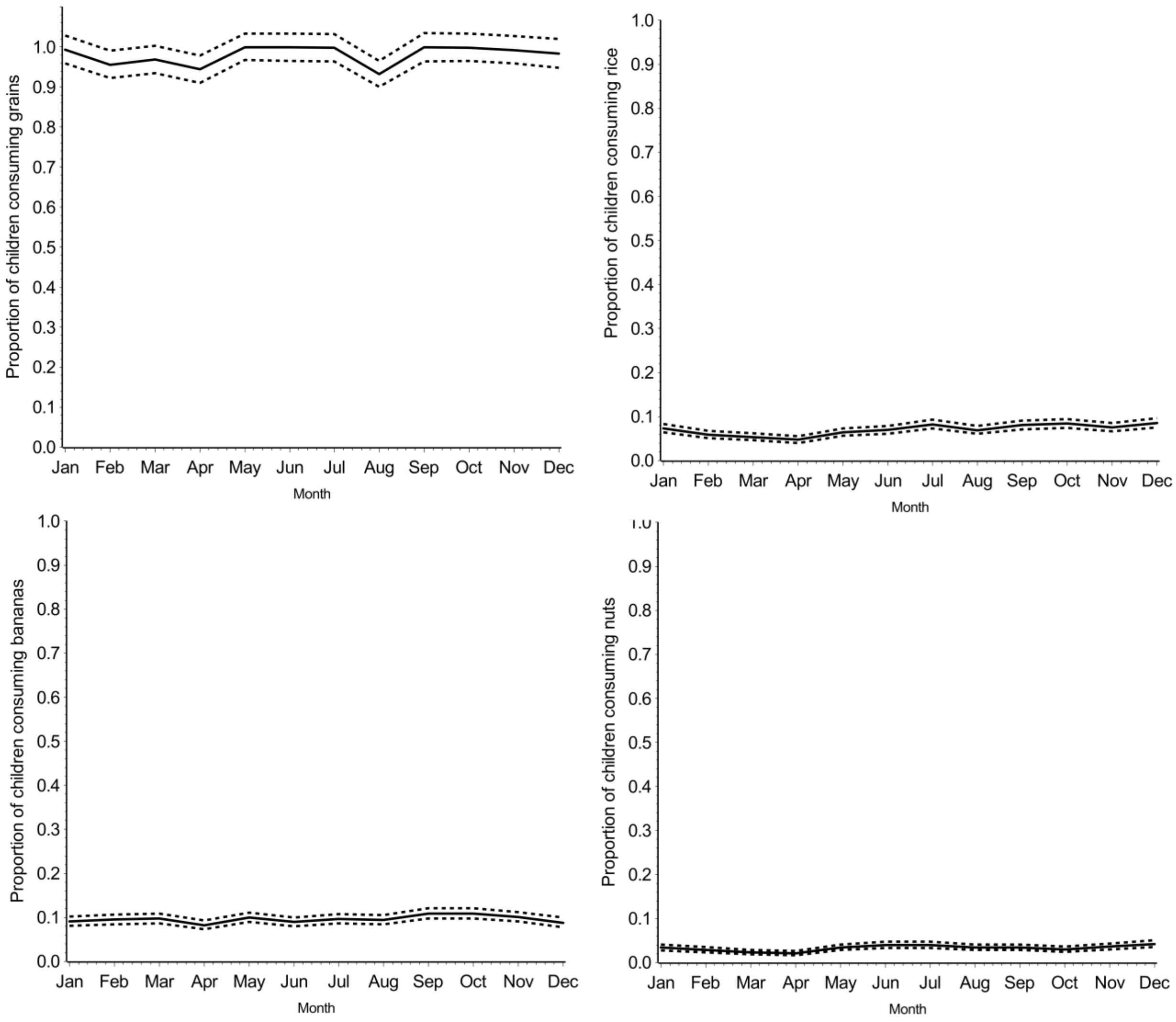

Figure S2. Child intake of non-seasonal foods by 24-hour food recall by calendar month among 262 children at the Haydom, Tanzania site of the MAL-ED study.

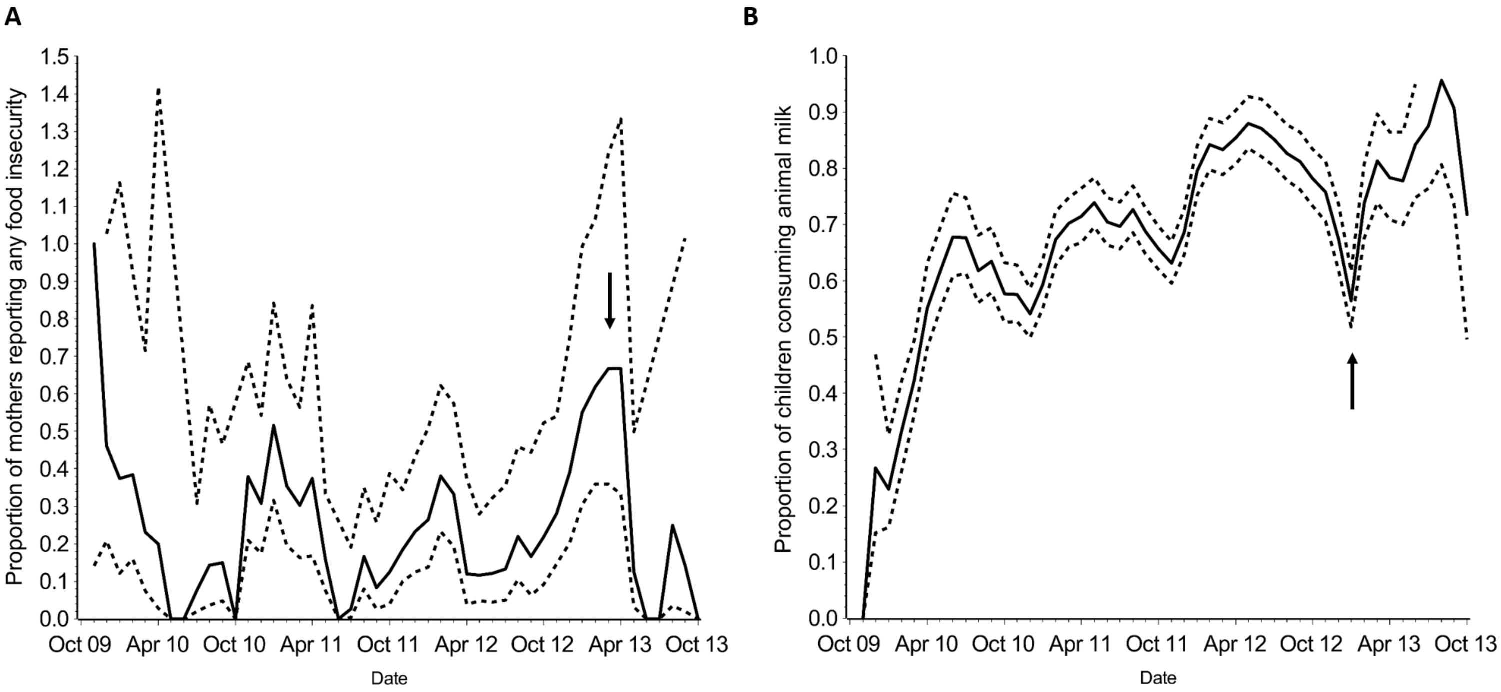

Figure S3. Proportion of mothers reporting food insecurity (A) and proportion of children consuming animal milk (B) by calendar month from 2009-2013 among 262 children at the Haydom, Tanzania site of the MAL-ED study. An example of acute correlation between lower animal milk consumption by 24-hour food recall and high food insecurity in January 2013 is indicated with arrows in both plots.

Note: The proportion of children consuming animal milk is low at the beginning of the study (October 2009 – April 2010) because children were enrolled with 17 days of birth, and most children were exclusively breastfed for the first few months of life.

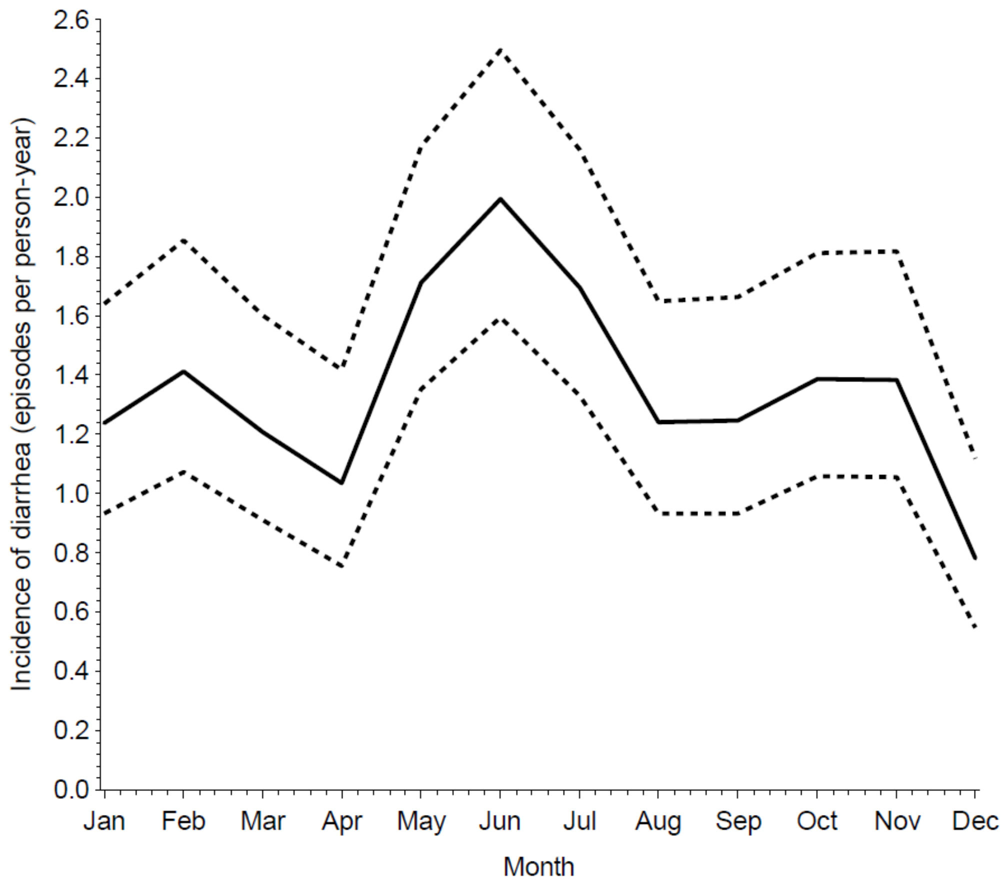

Figure S4. Incidence (solid line) and 95% confidence limits (dotted lines) of diarrhea by calendar month among 262 children at the Haydom, Tanzania site of the MAL-ED study.

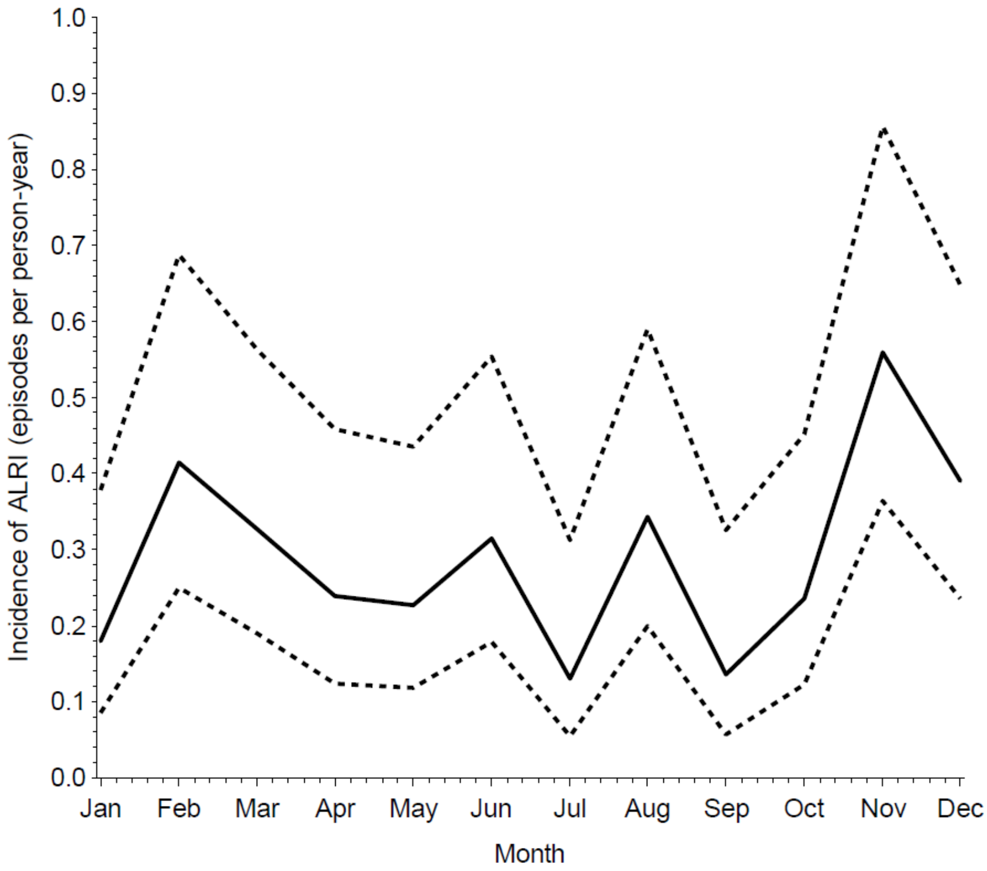

Figure S5. Incidence (solid line) and 95% confidence limits (dotted lines) of acute lower respiratory infections (ALRI) by calendar month among 262 children at the Haydom, Tanzania site of the MAL-ED study.

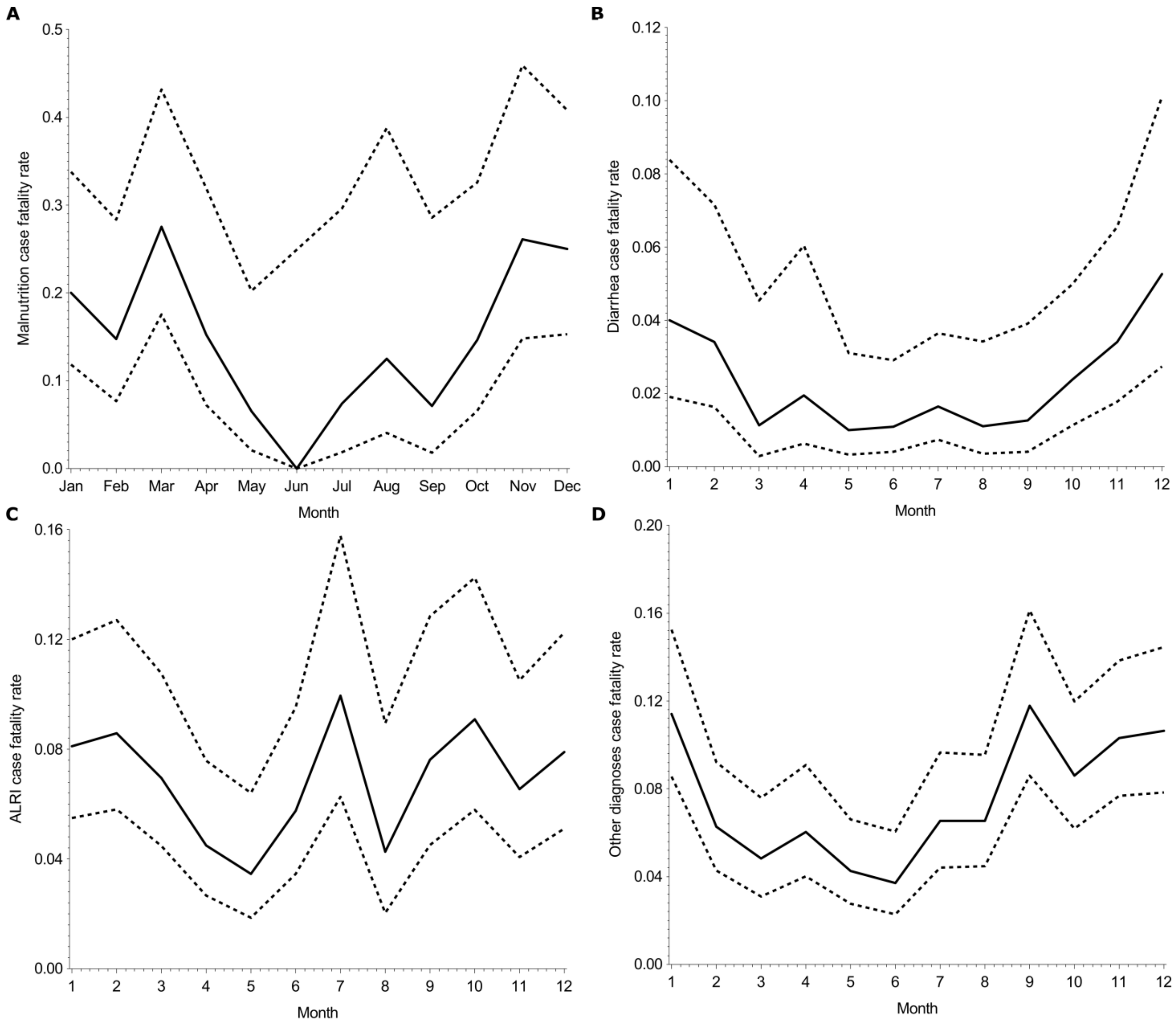

Figure S6. Seasonality of case fatality rates by diagnosis among children under 5 at Haydom Lutheran Hospital from 2010 to 2015. A: malnutrition; B: diarrhea; C: acute lower respiratory infections (ALRI); D: other.
